# Impact of biological manure substitution on grain yield, nitrogen recovery efficiency, and soil biochemical properties

**DOI:** 10.7717/peerj.17475

**Published:** 2024-05-28

**Authors:** Zhili Sun, Chengshun Wang, Jiabao Wang, Gang Wu, Manman Yuan, Haiming Zou, Yixiang Sun

**Affiliations:** 1 College Resource & Environment, Anhui Science & Technology University, Chuzhou, Anhui, China; 2 Institute of Soil and Fertilizer, Anhui Academy of Agricultural Sciences, Key Laboratory of Nutrient Cycling and Arable Land Conservation of An Hui Province, Hefei, Anhui, China

**Keywords:** N recovery efficiency, Soil biochemical property, Rice production, Biological manure fertilizer

## Abstract

Fertilization plays a crucial role in ensuring global food security and ecological balance. This study investigated the impact of substituting innovative biological manure for chemical fertilization on rice (*Oryza sativa* L) productivity and soil biochemical properties based on a three-year experiment. Our results suggested rice yield and straw weight were increased under manure addition treatment. Specifically, 70% of total nitrogen (N) fertilizer substituted by biological manure derived from straw, animal waste and microbiome, led to a substantial 13.6% increase in rice yield and a remarkable 34.2% boost in straw weight. In comparison to the conventional local farmer practice of applying 165 kg N ha^−1^, adopting 70% of total N plus biological manure demonstrated superior outcomes, particularly in enhancing yield components and spike morphology. Fertilization treatments led to elevated levels of soil microbial biomass carbon and N. However, a nuanced comparison with local practices indicated that applying biological manure alongside urea resulted in a slight reduction in N content in vegetative and economic organs, along with decreases of 10.4%, 11.2%, and 6.1% in N recovery efficiency (NRE), respectively. Prudent N management through the judicious application of partial biological manure fertilizer in rice systems could be imperative for sustaining productivity and soil fertility in southern China.

## Introduction

Agricultural practices play an essential contribution in global food security and ecosystem health, with fertilizer application being paramount. Traditionally, chemical nitrogen (N) fertilizers have been extensively utilized to enhance soil fertility and boost crop yields. However, concerns over associated ecological and environmental consequences have prompted researchers to explore alternative strategies (Smith et al., 2016; Doğan et al., 2023). Innovative approaches that promote sustainable agriculture, considering the intricate dynamics between soil health, crop yield, and environmental sustainability, are crucial for shaping the future of agriculture towards more eco-friendly and resilient practices (Tilman et al., 2017; Singh et al., 2023).

Chemical fertilizer is crucial for global food security and agricultural production. However, excessive use of chemical fertilizer could reduce grain yield and increase N loads to environment (Liu et al., 2024). Long-term use of chemical fertilizers could lead to soil acidification, salinization, and decreased microbial abundance, thereby impacting soil health (Musa et al., 2023). In addition, large amount use of chemical fertilizer poses the risk of water pollution, exacerbating degradation of aquatic ecosystems (Evans et al., 2019). To reduce chemical fertilizer to farmland, compost is one solution to improve crop growth and development and soil health.

Manure, a traditional and organic nutrient source, is gaining recognition as a promising alternative to chemical fertilizers (Ng et al., 2024). Biological manure, derived from organic sources such as animal waste and plant materials, has long been recognized for enhancing soil fertility and crop productivity. Compared to common manure, biological manure enriched with diverse microbiomes can target specific soil types and crops, thereby improving soil nutrients and moisture, and promoting increased crop productivity (Chaparro et al., 2012). Beyond supplying essential nutrients for crop development, its utilization presents the added benefit of recycling organic matter into the soil. As the agricultural paradigm undergoes transformations, it becomes crucial to comprehensively evaluate the ramifications of partial and complete substitution with manure applications (Hou et al., 2023). Understanding the holistic impact of transitioning to manure-based practices is essential for informed and sustainable agricultural decision-making, ensuring a balanced approach to nutrient management and soil health.

As global concerns about the environmental footprint of agriculture intensify, there is a paramount need to explore sustainable practices that balance productivity with ecological stewardship. The investigation into the partial substitution by manure applications represents a crucial step in evaluating the feasibility and consequences of adopting organic alternatives on a large scale. Thus, this study aims to elucidate the impacts of a novel biological manure application on grain yield, soil nutrients and microbial biomass in rice system during 2020–2022 in southern China.

## Materials and Methods

### Site description and experimental design

A three-year experiment was conducted to assess the impact of various N fertilization strategies on rice productivity in He County, Anhui province, China (N31.76828°, E118.20394°, elevation 18 m) from 2020 to 2022. The experimental site, characterized by a subtropical climate, had an average annual temperature of approximately 15.8 °C and annual precipitation of 1,067 mm. The predominant soil profile was hydragric paddy soil, equivalent to *Inceptisols* in the US soil classification system. Initial soil properties at depths of 0–20 cm in 2020 were as follows: pH 6.04, soil organic matter 14.8 g kg^−1^, total N content 1.07 g kg^−1^, available phosphate 36.6 mg kg^−1^ and available potassium 147 mg kg^−1^.

The study was carried out in a field trial initiated in June 2020 in a summer rice-winter wheat rotation that is the typical cropping system in southern China. The field experiment employed a randomized complete block design with plots measuring 60 m^2^ (6 m × 10 m). Three treatments were implemented in each season: the control treatment (N0) with 0 kg N ha^−1^; the conventional N treatment (N100) following local practices, applying 165 kg N ha^−^^1^; the manure plus 70% of the total 165 kg N ha^−1^ application (N70BM). Each experimental plot received 60 kg P_2_O_5_ of calcium superphosphate (12%) and 75 kg K_2_O ha^−1^ potassium sulfate (60%), and these fertilizers were incorporated into the soil at a depth of 10 cm for each season. The biological manure fertilizer, jointly developed by our experimental lab and Anhui Serte Fertilizer Co., Ltd, primarily utilized pig manure and straw as raw materials. It exhibited an effective viable count (Bacillus amyloliquefaciens SQR9) of ≥ 200 million CFU g^−1^, organic matter content of ≥ 50%, moisture content of ≤ 30%, and nutrient content of 15.6 g N kg^−1^, 26.1 g P_2_O_5_ kg^−1^, and 64.8 g K_2_O kg^−1^ in granular powder form. Plots were tilled before the crop was transplanted using a field cultivator to a depth of 10–15 cm for seed bed preparation. In a plot, rice was transplanted in mid-June and harvested in early October. Each treatment was replicated three times. The popular rice cultivar “Fuxiangzhan” was employed in this study. Field management practices, including tillage, weed, and pest control, were consistent with local farmer practices.

### Field sampling and analysis

Grain yield was determined by harvesting the whole plot area and weighing all leaves and stems for recording straw. Ten representative plants were sampled from the middle of each plot in each season when 80% of the plants reached physiological maturity to measure yield components: grain number per hectare, spike length, and kernel grain weight. The plant samples were oven dried to constant weight at 60 °C and then was measured N content in grain and straw by an elemental analyzer (Vario Max CN; Elemeta Analysensysteme GmbH, Hanau, Germany). Accumulated N content was calculated as the product of N content and dry matter weight.

In each season, soil samples were collected at a depth of 20 cm using the 5-point method, with three replications conducted after the rice harvest. The samples were processed by removing fine roots, gravel, and plant residues. Subsequently, the soil was divided into two parts: one served as air-drying soil through a 0.25 mm sieve for determining soil properties, while the other was stored at 4 °C for measuring soil NH_4_^+^ −N, NO_3_^−^ −N, microbial biomass C (MBC) and N (MBN) and soil enzyme activity. Soil NH_4_^+^ −N and NO_3_^−^ −N were measured by the continuous flow analyzer (FIAstar 5000 Analyzer; Foss Tecator, Hillerød, Denmark). Soil MBC and MBN were determined using the chloroform fumigation extraction method. Following fumigation, microbial residues are extracted, and organic C and N are quantified (Vance, Brookes & Jenkinson, 1987).

Soil catalase activity was evaluated using the potassium permanganate titration method (Guan, Zhang & Zhang, 1986; Wang et al., 2020). In this process, a soil sample (2 g) was mixed with 40 ml of distilled water and 5 ml of hydrogen peroxide (3%), shaken for 30 min, and then filtered. A 25 ml filtrate was titrated to a pink color endpoint using 0.1 M potassium permanganate.

Urease activities were determined following the method outlined by Guan, Zhang & Zhang (1986) and Yin et al. (2014). In this procedure, soil samples (2 g) were treated with 10 ml urea solution (10%), 20 ml of citrate buffer (1 M, pH 6.7), and 1 ml of methylbenzene. After incubating at 37 °C for 24 h, the solution was filtered, and 1 ml of filtrate was mixed with 20 ml of distilled water, 4 ml of sodium phenolate hydroxide, and 3 ml of sodium hypochlorite. NH_4_^+^ −N was then analyzed using a spectrophotometer at 578 nm after 20 min. Urease activity was expressed as milligrams of NH_4_^+^ −N per gram of dry soil released in 24 h.

The invertase activity was assessed following the protocols outlined by Guan, Zhang & Zhang (1986). In this procedure, invertase activity was measured using 3,5-dinitrosalicylic acid as the reagent and sucrose solution as the substrate. The results were expressed as the mass (mg) of glucose per gram of soil after a 24-hour incubation period.

Apparent N recovery efficiency (NRE) was calculated by N concentration and plant biomass as defined by a previous study (Conant, Berdanier & Grace, 2013).

(1) \begin{eqnarray*}\mathrm{NRE}= \frac{\mathrm{N}~{\text{uptake}}_{(\text{fertilized})}-\mathrm{N}~{\text{uptake}}_{(\text{none}-\text{fertilized})}}{\text{amount of}~\mathrm{N}~\text{applied}} .\end{eqnarray*}

### Statistical analysis

To assess the differences in grain yield, yield components, biomass, and soil properties between the various N treatments (*i.e.,* N0, N100, and N70BM) and experimental year, two-way analysis of variance (ANOVA) and least significant difference (LSD) tests (*P* < 0.05) was performed using SPSS 20.0 software (2011: SPSS, Inc., Chicago, IL, USA). The detailly statistical results were presented in the Table S1.

## Result

### Grain yield and straw weight

**Figure 1:**
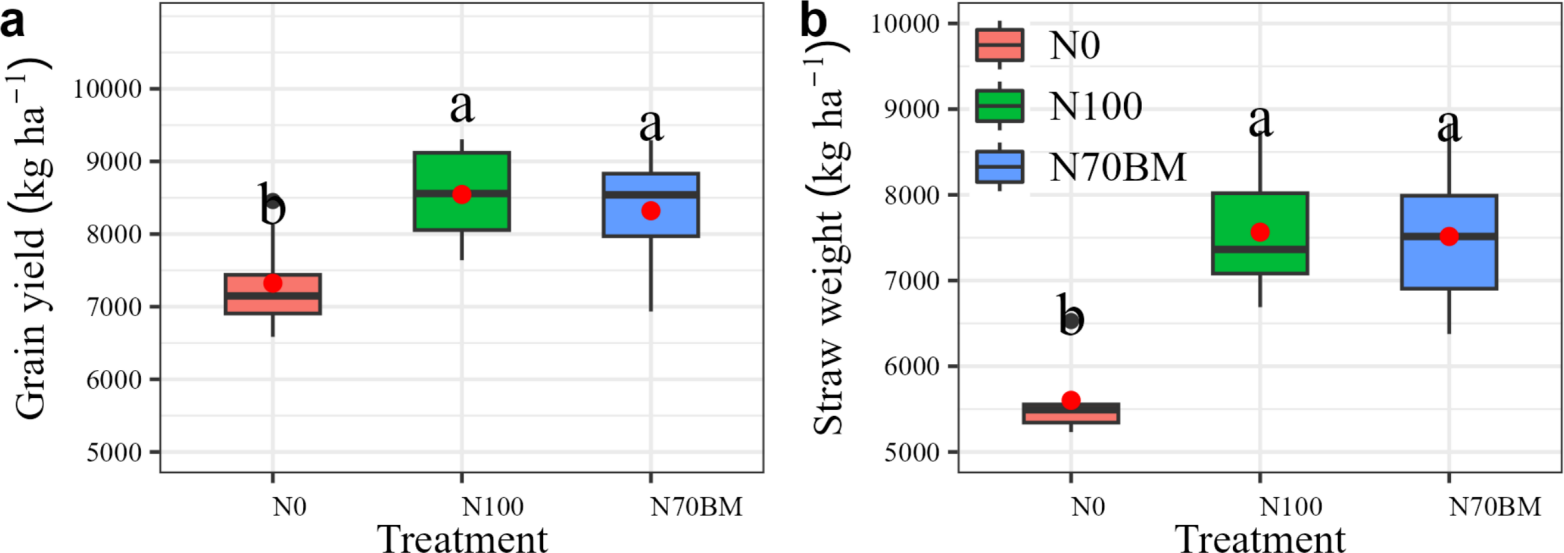
Boxplots summarizing variation in rice grain yield (A) and straw weight (B) under different fertilizer treatments between 2020 and 2022. For each boxplot, the central mark is the median, the red square indicates the mean value, the edges of the box are the 25th and 75th percentiles, and the whiskers extend to the extreme data points not considered to be outliers. Different lowercase letters represent significant differences (*P* < 0.05) between the different N treatments.

Grain yield and straw weight showed significant differences under fertilizer treatments (Fig. 1). Compared with N0, 100% N application (N100) increased the average yield by 1,221 kg ha^−1^ and straw weight by 1,913–1,963 kg ha^−1^, respectively (*P* < 0.05, Table S1). Substituting manure for 30% of total N amount (N70BM) increased the average yield by 13.6% and straw weight by 34.2%, respectively (*P* < 0.05, Table S1). However, both grain yield and straw weight under 30% of total N substituted by manure were slightly smaller than those under 100% of total N application. Furthermore, the variations in year effects and their interactions with N management treatments did not yield significant impacts on grain yield or other measured traits (Table S1). Consequently, these factors were not included in the subsequent analysis of results.

### N recovery efficiency and N uptake in plant

**Figure 2:**
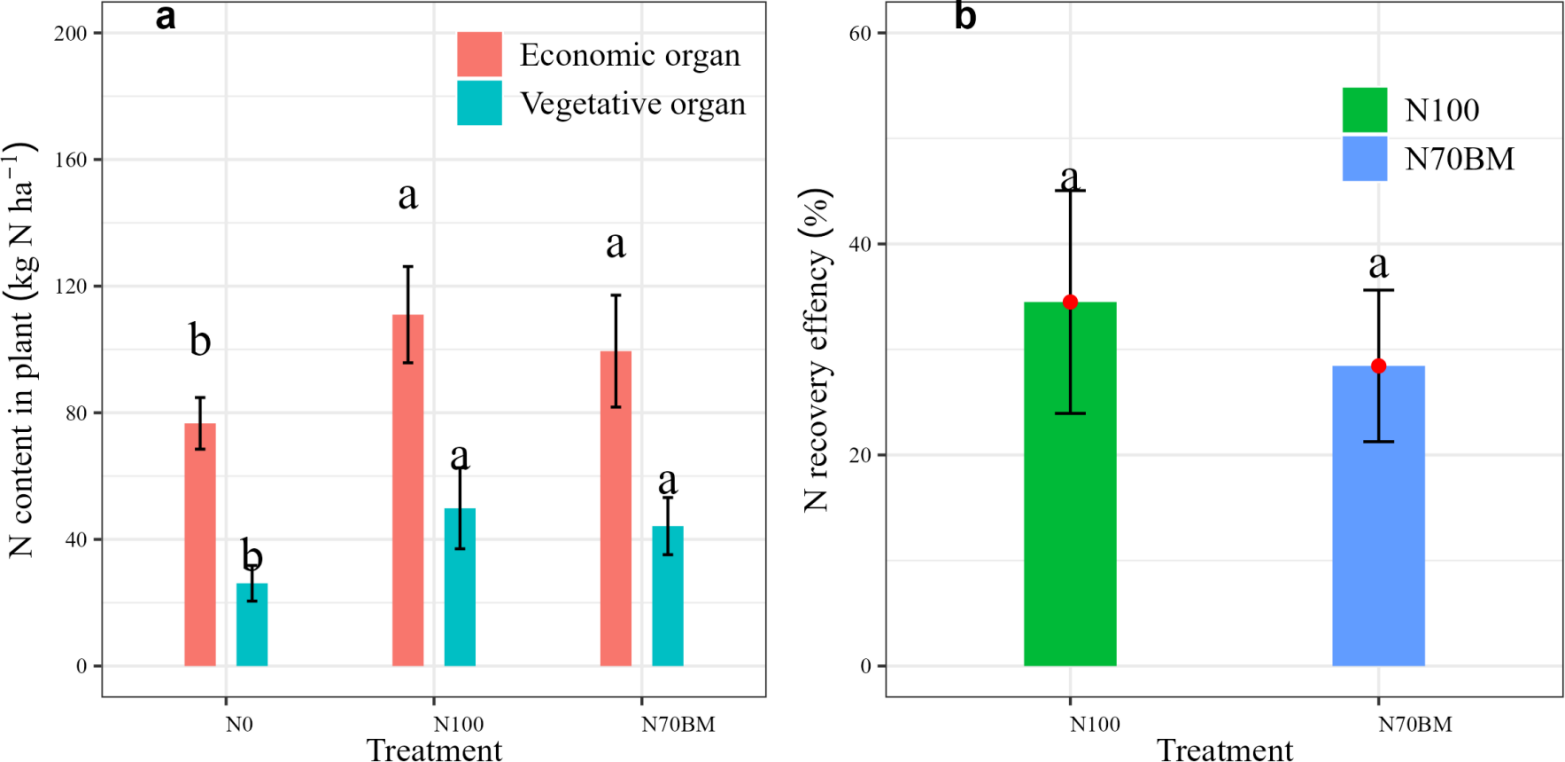
Variations of N uptake between vegetative and economic organs (A) and NRE at harvest among different N treatments during 2020 –2022. The error bar represents the standard deviation of each index in each treatment. Different lowercase letters represent significant differences (*P* < 0.05) between the different N treatments.

Fertilization regime increased N content in economic and vegetative organs (*P* < 0.05, Fig. 2A, Table S1) and N recovery efficiency (*P* > 0.05, Fig. 2B). Specifically, N content in grain yield and straw at harvest was highest under N100 treatment, at 111 kg N ha^−1^ and 49.8 kg N ha^−1^ respectively. Under N70BM treatment, N content in plant was slightly lower by 10.4% and 11.2% compared to N100 (*P* > 0.05), respectively (Fig. 2A). Similarly, N recovery efficiency under N100 treatment was slightly higher by 6.1% compared to N70BM treatment (*P* > 0.05, Fig. 2B).

### Yield components and N uptake

Fertilization regime influenced yield components and N concentrations in grain and straw (Table 1). Specifically, there was no significant difference in spike length, kernel grain weight, grain number and N concentration in straw between fertilized and unfertilized treatments. However, the average effective spike and N concentration in grain among N management treatments were 20.6–21.6% and 2.3–14.5% higher than that under N0, respectively. Overall, the N70BM treatment showed a slight advantage in increasing spike length, kernel grain weight and grain number, while the N100 treatment had potential to enhance effective spike and N concentration in plants compared with other treatments.

**Table 1:** Changes of yield components and N concentrations in grain and straw at harvest during 2020–2022.

Treatment	Spike length (cm)	Effective spike (×10^5^ ha^−1^)	Kernel grain weight (g^−1^)	Grain number (spike^−1^)	Straw N content (g kg^−1^)	Grain N content (g kg^−1^)
N0	26.1 ± 3.7a	16 ± 3.1b	22.4 ± 0.8a	164.3 ± 15.8a	5.2 ± 1.3a	11.0 ± 1.0b
N100	26.5 ± 3.6a	19.6 ± 2.5a	22.4 ± 1.1a	167.2 ± 28.6a	6.0 ± 1.6a	12.6 ± 1.2a
N70BM	27.6 ± 1.6a	19.3 ± 4.9a	22.5 ± 0.6a	169.9 ± 15.2a	5.8 ± 0.6a	11.3 ± 1.3ab

### Soil biochemical property

#### Soil nutrient and microbial biomass

**Figure 3:**
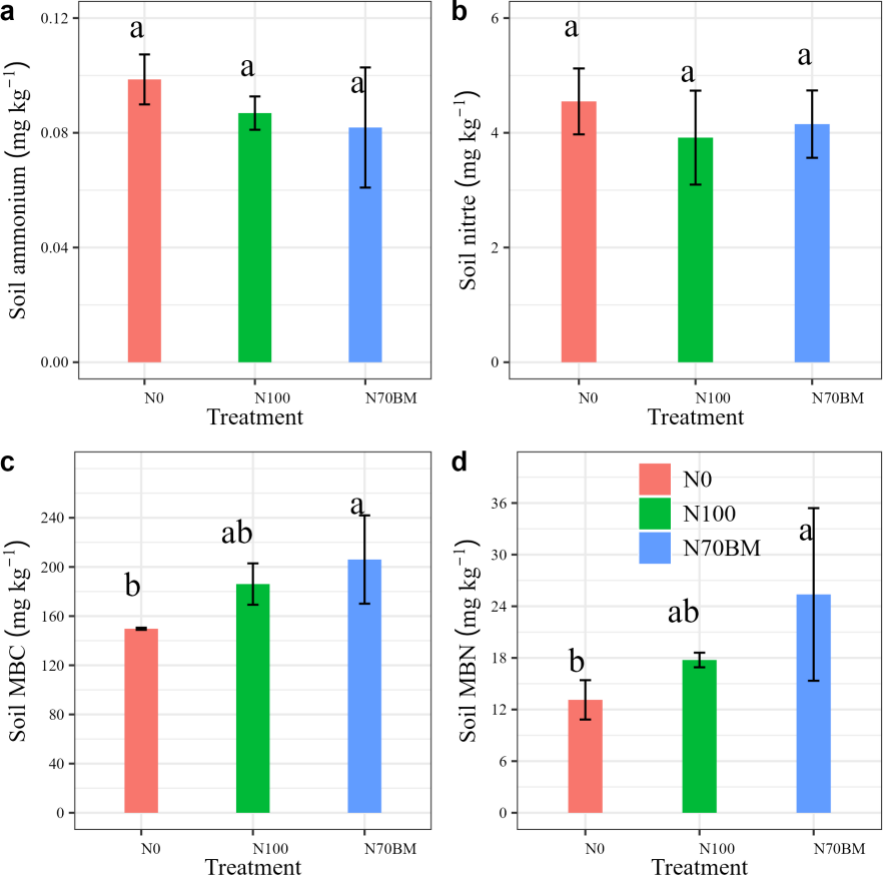
Average soil ammonium, nitrate (A, B) and microbial biomass C/N (C, D) in each treatment from 2020 to 2022. The error bar represents the standard deviation of each index in each treatment. Different lowercase letters represent significant differences (*P* < 0.05) between the different N treatments.

Soil nutrient and microbial biomass at harvest for different fertilization regime are shown in Fig. 3. The average soil ammonium and nitrate N contents varied from 0.06–0.1 g kg^−1^ and 3.0–5.2 g kg^−1^ under all treatments, respectively (Figs. 3A and 3B). The lowest ammonium N content was observed under N70BM while the lowest nitrate N content was observed under N100, with an average value of 0.08 g kg^−1^ and 3.9 g kg^−1^, respectively. However, compared to N0 treatment, average soil ammonium and nitrate N content slightly decrease by 0.02 g kg^−1^ and 0.5 g kg^−1^ among the fertilization treatments.

Soil microbial biomass C (MBC) and N (MBN) were generally higher in applied N treatments than in the zero N treatment. The largest MBC and MBN occurred in N70BM, while the lowest MBC and MBN occurred in N0 treatment. N fertilization significantly affected MBC and MBN, which showed an increase by 24.3–37.3% and 35.5–93.3% for the MBC and MBN in response to N fertilization (*P* < 0.05, Fig. 3C and Table S1).

#### Soil enzyme activity

**Figure 4:**
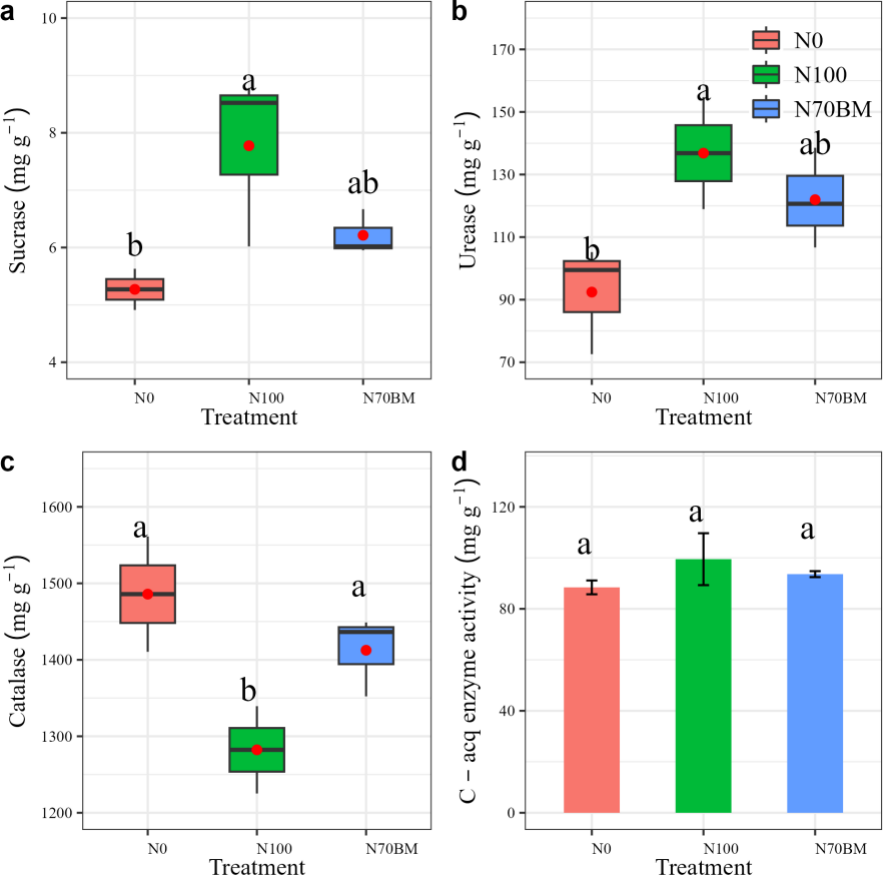
(A–D) Boxplots summarizing variation in soil sucrase, urease and catalase under different fertilizer treatments between 2020 and 2022. For each boxplot, the central mark is the median, the red point indicates the mean value, the edges of the box are the 25th and 75th percentiles, and the whiskers extend to the extreme data points not considered to be outliers. The error bar represents the standard deviation of each index in each treatment. Different lowercase letters represent significant differences (*P* < 0.05) between the different N treatments.

The soil enzyme activities varied among fertilization regimes (Fig. 4). High N addition resulted in the largest soil sucrase and urease enzyme activities, involved in C and N cycling, which were 17.8% to 47.6% and 31.9% to 48.1% higher than those from other treatments, respectively (*P* < 0.05, Figs. 4A and 4B, Table S1). The lowest catalase enzyme activity was observed under N100 treatment (*P* < 0.05, Fig. 4C, Table S1). Similarly, the highest C-acquisition enzyme activities occurred under N100 treatment (*P* > 0.05, Fig. 4D).

## Discussion

### Fertilizer regime impacts on grain yield, its components and N uptake

Previous studies have demonstrated that appropriate N management can enhance plant growth, grain yield, and its components (Makino, Suzuki & Ishiyama, 2022). Grain yield, a key indicator of agricultural productivity, depends on the intricate interplay between yield components and N remobilization to economic organs in plants. Unraveling these connections is essential for maximizing grain yield while minimizing N losses to environment (Long et al., 2006; Liu et al., 2020). Our study revealed that proper N management through urea or biological manure significantly increased grain yield and N uptake in economic organs of plant, with the magnitude ranging from 7,640 kg ha^−1^ to 9,305 kg ha^−1^ and from 99 kg ha^−1^ to 131 kg ha^−1^, respectively (Figs. 1 and 2). This finding aligns with previous research indicating that fertilization substantially enhances biomass production by improving photosynthesis efficiency and facilitating N mobilization to leaves and stems in plants (Yoon et al., 2020; Makino, Suzuki & Ishiyama, 2022). However, compared to the N100 treatment, substitution with biological manure fertilizer slightly reduced grain yield, its components and accumulated N in plant. Nutrients are gradually released from biological manure fertilizer, promoting plant growth and fulfilling N requirements, thereby minimizing mineral N losses (Qaswar et al., 2020). Moreover, a modest increase in spike length, kernel grain weight and grain number per spike was observed under N70BM compared to single N fertilization (Table 1). The gradual release of nutrients from organic manure enhanced the coordination of N supply and expedited the transfer of N from leaves and stems to economic organs during the later stages of growth. This is associated with the difference in assimilation distribution between tiller and main shoot due to the response of tiller inhibition gene to different N type (Duggan et al., 2005; Moeller & Rebetzke, 2017). Additionally, our study showed that the similar magnitude and pattern for grain yield and N uptake were observed among different experimental year (Table S1). This agrees with a previous study reported that temperatures and precipitation have less effects on irrigated rice yield (Bannayan & Sanjani, 2011).

### Fertilization regime effects on soil nutrient, microbial biomass and enzymes

Of the soil nutrients involved in this study, soil ammonium and nitrate consistently exhibited the most uniform responses across different treatments (Figs. 3A and 3B). Soil nutrient content under fertilization was slightly lower compared to plots with zero N application. The increased fertilizer application may have facilitated a more efficient uptake of nutrients by crops, leading to enhanced crop yield and nutrient absorption despite the slight decrease in soil nutrient content (Li et al., 2019). This is consistent with the notion that the additional nutrients provided through fertilization compensated for the marginal decrease in soil nutrient levels, thereby contributing to improved crop performance and nutrient utilization (Govindasamy et al., 2023).

Organic manure increased soil microbial biomass compared to sole chemical nitrogen addition (Fig. 4), likely due to the enhanced availability of organic carbon and nutrients (Zhang et al., 2020). The substantial application of manure application in soil acts as a source of substrates and energy, thereby promoting microbial growth and activity (Ling et al., 2022). Our findings indicate that both MBC and MBN were elevated under N70BM compared to sole chemical N treatment (Figs. 3C and 3D). This aligns with prior research indicating that abundant organic materials integrated into soil can supply ample nutrients for microbial metabolism, thereby stimulating microbial growth and enzyme secretion, particularly catalase (Zheng et al., 2019; Wang et al., 2022; Fig. 4C).

Past studies in paddy soil have indicated that soil enzyme activity can be enhanced by N amendments (Xiao, He & Wang, 2023 [urease and phosphatase]; (Li et al., 2022) [*β*-1,4-N-acetylglucosaminidase]; Sharma et al., 2021 [urease and L-asparaginase]; Hu et al., 2023 [*β*-1,4-glucosidase, *β*-N-acetyl-glucosaminidase and phenol oxidase]). Consistent with these findings, our results demonstrate that soil urease and sucrase activity increased under both N100 and N70BM treatments. The elevation in urease activity facilitates the conversion of soil organic N into ammonium N through mineralization, thereby enhancing its availability for plant uptake (Liang et al., 2003). However, soil catalase activity may be repressed by fertilization, potentially due to alterations in soil microbial communities and their metabolic activities induced by fertilization (Zheng et al., 2019). Interestingly, our results indicate a decrease in urease and sucrase activity in paddy soil, while catalase decreased with biological manure compared to chemical N addition (Fig. 4). The observed phenomenon may be attributed to the release of substances by organic manure, resulting in an increase in the soil C/N ratio (Li et al., 2018; Yang et al., 2023). Such alterations in C/N ratio could prompt shifts in soil microbial communities, considering the presence of both organic and inorganic N forms (Luo et al., 2016; Wu et al., 2020), thereby influencing urease and sucrase enzyme activities. However, it is crucial to note that catalase activity may exhibit distinct responses in the context of these alterations. To elucidate the effect of biological manure on soil C acquisition enzyme activity, more work should be made in the future.

## Conclusion

Our study underscores the vital role of proper N management, particularly through the incorporation of biological manure, in significantly impacting grain yield, its components, and nitrogen remobilization within plants, all of which are pivotal for enhancing agricultural productivity. Substituting biological manure for chemical fertilizers markedly boosts rice yield and straw weight. Specifically, the application of 70% total chemical N combined with 2,000 kg ha^−1^ of biological manure is superior to local practices, notably in maintaining rice yield and augmenting yield components, as well as in enhancing soil microbial biomass. Fertilization practices exerted a discernible influence on soil ammonium and nitrate content, with slightly lower nutrient levels observed in this study, yet efficiently compensated by enhanced nutrient uptake. Prudent N management, particularly the judicious integration of partial biological manure, emerges as indispensable for sustaining both productivity and soil fertility in southern China.

##  Supplemental Information

10.7717/peerj.17475/supp-1 Supplemental Information 1 Raw Data

10.7717/peerj.17475/supp-2 Supplemental Information 2 Supplementary tables A correlation analysis of the data.

## Additional Information and Declarations

### Data Availability

The following information was supplied regarding data availability: The raw data is available in the Supplementary File.
