# Supplementary material for: Impact of biological manure substitution on grain yield, nitrogen recovery efficiency, and soil biochemical properties
Source: PeerJ. 2024 May 28;12:e17475. doi: 10.7717/peerj.17475 (PMC11141546; doi:10.7717/peerj.17475)
Supplement: Supplemental Information 2 — A correlation analysis of the data. [file peerj-12-17475-s002.docx]

**Appendix for**

***Impact of biological manure substitution on grain yield, nitrogen recovery efficiency, and soil biochemical properties***

*Zhili Sun ^1^, Chengshun Wang ^1^, Jiabao Wang ^2^, Gang Wu ^2^, Manman Yuan ^2^, Haiming Zou ^1*^, Yixiang Sun ^2*^*

*^1^ Anhui Science & Technology University, College Resource & Environment, Donghua Rd 9, Chuzhou 233100, China*

*^2^ Key Laboratory of Nutrient Cycling and Arable Land Conservation of An Hui Province, Institute of Soil and Fertilizer, Anhui Academy of Agricultural Sciences, Hefei 230001, China*

*^*^ Corresponding author: Yixiang Sun and Haiming Zou*

*Email: sunyixiang@aaas.org.cn and zouhm@ahstu.edu.cn*

Table S1. Two-way ANOVA and LSD tests for the effects of fertilization treatments and experimental year on average crop yield, yield components, biomass and soil biochemical property from 2020 to 2022 (***, *P* < 0.001; **, *P* < 0.01; *, *P* < 0.05).

|  | Df | MS | F | *P* | Sig |  |  | Df | MS | F | *P* | Sig |
| --- | --- | --- | --- | --- | --- | --- | --- | --- | --- | --- | --- | --- |
| Grain yield | | | | | |  | Total N uptake | | | | | |
| Year | 1 | 59832 | 0.2 | 0.689 |  |  | Year | 1 | 154 | 0.6 | 0.466 |  |
| Treatment | 2 | 5987053 | 16.5 | <0.001 | *** |  | Treatment | 2 | 5634 | 20.8 | <0.001 | *** |
| Year×Treatment | 2 | 365105 | 1.0 | 0.384 |  |  | Year:Treatment | 2 | 122 | 0.5 | 0.647 |  |
| Straw weight | | | | | |  | Sucrase | | | | | |
| Year | 1 | 443545 | 0.7 | 0.416 |  |  | Year | 1 | 1 | 0.9 | 0.403 |  |
| Treatment | 2 | 7511769 | 12.0 | 0.001 | ** |  | Treatment | 2 | 5 | 3.7 | 0.155 |  |
| Year×Treatment | 2 | 87557 | 0.1 | 0.870 |  |  | Year×Treatment | 2 | 0 | 0.0 | 0.954 |  |
| Spike length | | | | | |  | Urease | | | | | |
| Year | 1 | 99 | 32.2 | 0.000 | *** |  | Year | 1 | 66 | 0.1 | 0.742 |  |
| Treatment | 2 | 3 | 1.1 | 0.355 |  |  | Treatment | 2 | 1533 | 3.0 | 0.189 |  |
| Year×Treatment | 2 | 6 | 1.8 | 0.207 |  |  | Year×Treatment | 2 | 91 | 0.2 | 0.843 |  |
| Effective spikes | | | | | |  | Catalase | | | | | |
| Year | 1 | 36 | 5.4 | 0.039 | * |  | Year | 1 | 3084 | 2.0 | 0.249 |  |
| Treatment | 2 | 42 | 6.3 | 0.014 | * |  | Treatment | 2 | 31887 | 21.0 | 0.017 | * |
| Year×Treatment | 2 | 22 | 3.3 | 0.071 | . |  | Year×Treatment | 2 | 7884 | 5.2 | 0.106 |  |
| Kernel grain weight | | | | | |  | Soil enzyme | | | | | |
| Year | 1 | 8 | 43.1 | <0.001 | *** |  | Year | 1 | 44 | 0.9 | 0.402 |  |
| Treatment | 2 | 0 | 0.1 | 0.890 |  |  | Treatment | 2 | 92 | 2.0 | 0.284 |  |
| Year×Treatment | 2 | 0 | 2.5 | 0.126 |  |  | Year×Treatment | 2 | 20 | 0.4 | 0.683 |  |
| Grain number | | | | | |  | Ammonium | | | | | |
| Year | 1 | 3073 | 12.8 | 0.004 | ** |  | Year | 1 | 0 | 0.0 | 0.867 |  |
| Treatment | 2 | 49 | 0.2 | 0.820 |  |  | Treatment | 2 | 0 | 1.3 | 0.389 |  |
| Year×Treatment | 2 | 275 | 1.1 | 0.351 |  |  | Year×Treatment | 2 | 0 | 1.7 | 0.316 |  |
| N content in vegetative organs | | | | | |  | Nitrate | | | | | |
| Year | 1 | 0 | 0.1 | 0.727 |  |  | Year | 1 | 0 | 0.4 | 0.568 |  |
| Treatment | 2 | 0 | 0.7 | 0.531 |  |  | Treatment | 2 | 0 | 0.5 | 0.674 |  |
| Year×Treatment | 2 | 0 | 0.4 | 0.659 |  |  | Year×Treatment | 2 | 0 | 0.3 | 0.769 |  |
| N content in economic organs | | | | | |  | MBC | | | | | |
| Year | 1 | 0 | 1.0 | 0.329 |  |  | Year | 1 | 1294 | 3.8 | 0.147 |  |
| Treatment | 2 | 0 | 3.0 | 0.090 | . |  | Treatment | 2 | 2446 | 7.2 | 0.072 | . |
| Year×Treatment | 2 | 0 | 0.1 | 0.896 |  |  | Year×Treatment | 2 | 415 | 1.2 | 0.411 |  |
| N uptake in vegetative organs | | | | | |  | MBN | | | | | |
| Year | 1 | 100 | 1.5 | 0.237 |  |  | Year | 1 | 24 | 0.4 | 0.553 |  |
| Treatment | 2 | 919 | 14.3 | 0.001 | *** |  | Treatment | 2 | 115 | 2.1 | 0.264 |  |
| Year×Treatment | 2 | 256 | 4.0 | 0.048 | * |  | Year×Treatment | 2 | 14 | 0.3 | 0.783 |  |
| N uptake in economic organs | | | | | |  |  |  |  |  |  |  |
| Year | 1 | 96 | 0.4 | 0.532 |  |  |  |  |  |  |  |  |
| Treatment | 2 | 1832 | 7.9 | 0.006 | ** |  |  |  |  |  |  |  |
| Year×Treatment | 2 | 88 | 0.4 | 0.692 |  |  |  |  |  |  |  |  |
